# The Odorant Receptor Co-Receptor from the Bed Bug, *Cimex lectularius* L

**DOI:** 10.1371/journal.pone.0113692

**Published:** 2014-11-20

**Authors:** Immo A. Hansen, Stacy D. Rodriguez, Lisa L. Drake, David P. Price, Brittny N. Blakely, John I. Hammond, Hitoshi Tsujimoto, Erika Y. Monroy, William A. Maio, Alvaro Romero

**Affiliations:** 1 Department of Biology, New Mexico State University, Las Cruces, New Mexico, United States of America; 2 Department of Entomology, Plant Pathology and Weed Science, New Mexico State University, Las Cruces, New Mexico, United States of America; 3 Department of Biology, University of New Mexico, Albuquerque, New Mexico, United States of America; 4 Department of Chemistry & Biochemistry, New Mexico State University, Las Cruces, New Mexico, United States of America; 5 Molecular Biology Program, New Mexico State University, Las Cruces, New Mexico, United States of America; Monell Chemical Senses Center, United States of America

## Abstract

Recently, the bed bug, *Cimex lectularius* L. has re-emerged as a serious and growing problem in many parts of the world. Presence of resistant bed bugs and the difficulty to eliminate them has renewed interest in alternative control tactics. Similar to other haematophagous arthropods, bed bugs rely on their olfactory system to detect semiochemicals in the environment. Previous studies have morphologically characterized olfactory organs of bed bugs’ antenna and have physiologically evaluated the responses of olfactory receptor neurons (ORNs) to host-derived chemicals. To date, odorant binding proteins (OBPs) and odorant receptors (ORs) associated with these olfaction processes have not been studied in bed bugs. Chemoreception in insects requires formation of heteromeric complexes of ORs and a universal OR coreceptor (Orco). Orco is the constant chain of every odorant receptor in insects and is critical for insect olfaction but does not directly bind to odorants. Orco agonists and antagonists have been suggested as high-value targets for the development of novel insect repellents. In this study, we have performed RNAseq of bed bug sensory organs and identified several odorant receptors as well as Orco. We characterized Orco expression and investigated the effect of chemicals targeting Orco on bed bug behavior and reproduction**.** We have identified partial cDNAs of six *C. lectularius* OBPs and 16 ORs. Full length bed bug Orco was cloned and sequenced. Orco is widely expressed in different parts of the bed bug including OR neurons and spermatozoa. Treatment of bed bugs with the agonist VUAA1 changed bed bug pheromone-induced aggregation behavior and inactivated spermatozoa. We have described and characterized for the first time OBPs, ORs and Orco in bed bugs. Given the importance of these molecules in chemoreception of this insect they are interesting targets for the development of novel insect behavior modifiers.

## Introduction

Bed bugs, *Cimex lectularius* L. (Hemiptera: Cimicidae), are obligate hematophagous insects that have become a serious and growing global problem in the last decade [1]–[5]. Although they are not known to be vectors of human diseases, bed bugs have severe adverse effects on health and quality of life. Bites of bed bugs can produce several skin clinical syndromes including severe bullous reactions that resemble the Churg-Strauss syndrome [6]–[8]. Chronic blood loss and iron-deficiency anemia have also been reported in people who have been continuously exposed to severe bed bug infestations [9]–[11]. Bed bugs can also create anxiety, and people who are repeatedly bitten may develop nervous behavior, agitation, stress and sleeplessness [12]–[14]. The adverse effects of bed bugs on humans have led the Environmental Protection Agency and Centers for Disease Control and Prevention to consider this pest of significant public health importance [15].

Control of bed bugs is primarily based on intensive application of a limited number of insecticides, mainly pyrethroids [16]–[18]. Heavy reliance of chemical insecticides has selected for resistance in bed bug populations worldwide [4], [19]–[24]. The high incidence of insecticide resistance and failure to eliminate resistant bed bugs is a contributing factor for the spread of this pest [21]. Therefore, alternative effective methods for bed bug control are of great importance [25]. For development of such control methods is neccesary to increase our knowledge in the biology and behavior of this pest.

Bed bugs have nocturnal habits and during day-time, they remain hidden and aggregated in cracks or crevices.This state of immobility is induced by aggregation pheromones present in frass and body secretions [26]–[30]. At night, when host activity is minimal, bed bugs leave their harborages in search of a blood meal. Onset of nocturnal locomotor activity in bed bugs is driven by hunger but it is controlled by a circadian clock [29]. As many other blood feeding arthropods, bed bugs rely on their senses to locate a host by using a combination of heat and kairomones [31]. Carbon dioxide has been found to be the most attractive to bed bugs and it has been shown to have an additive effect when used with heat [32], [33]. Morphological studies show that bed bugs bear olfactory-like sensilla in their antennae distributed along the four antennal segments which confirm their importance as olfactory regions of bed bugs [34]. Adult female *Aedes aegypti* mosquitoes have roughly 2000 olfactory sensilla on each antenna flagellum and olfactory clues play a critical role in mosquito host-finding behavior [35].

Odorants are thought to interact with at least two different classes of insect proteins when entering an olfactory sensillum: odorant-binding proteins (OBPs) and odorant receptors (ORs) [36]. OBPs facilitate the transport of odor molecules through the sensillum lymph to the OR proteins that are located in dendrites of olfactory neurons [36]. A battery of different ORs, expressed exclusively in single olfactory neurons, confer the specificity of odor reception. Insect odorant receptors are heteromeric complexes that have a constant and a variable chain [36]. The variable chain binds the odor and is responsible for specificity [36]. Odorant receptor co-receptor (Orco) is the constant chain. Upon binding an odor molecule OR/Orco complexes mediate cation influx directly or through other signal transduction pathways [36]. Orco has been suggested as a novel high-value target for the development of a new class of insect repellents and both antagonist and agonist have been identified [37], [38].

In addition to olfactory organs, Orco is expressed in spermatozoa of mosquitoes and other insects. A recent study by Pitts et al. [39] showed that sperm of the malaria vector mosquito, *Anopheles gambiae* and the dengue mosquito, *Ae. aegypti* were activated in the presence of an Orco agonist, VUAA1, while this effect was inhibited by addition of the Orco antagonist VU0183254 (VUANT in [39]). These authors also found Orco protein expression in sperm of other insect species (the fruit fly, *Drosophila melanogaster*, the jewel wasp, *Nasonia vitripennis* and the Asian tiger mosquito, *Aedes albopictus*), which suggested that Orco expression (and potentially function) in sperm is conserved across different insect orders [39]. Here we show expression of Orco in antennae, sperm and other tissues, and that the Orco agonist VUAA1 has a different effect on bed bug sperm compared to its effect on sperm of mosquitoes.

## Materials and Methods

### Insect culture

Bed bugs were maintained at 25°C, 65±5% RH, and a photoperiod of 14∶10 (L:D) h. The bed bug colony was collected from a human dwelling in New Jersey City, NJ. Bed bugs were collected by a pest control company and the resident or owner of the property gave permission to collect bed bugs from the site. Insects were fed with a parafilm-membrane feeder containing defibrinated citrated rabbit blood (Quad Five, Ryegate, MT) that was heated to 39°C with a circulating water bath [40]. Adult male bed bugs used in behavioral bioassays were unfed for maximum of a week.

### Illumina Library Preparation and Sequencing

Labia, antenna, maxillae and mandibles were dissected from 50 males and 50 females, placed in Trizol and stored at –80°C until ready for use. Total RNA was then extracted from these samples according to the W.M. Keck Foundation protocol. A Nanodrop 1000 (Thermo scientific, Waltham, MA) was used to quantify total RNA concentration. Starting RNA quality was assessed visually by an RNA gel. RNA was pooled to approximately 500 nanograms of total RNA and was then used to prepare a library using the Illumina TruSeq RNA Sample Preparation Kit v2 according to the manufacturer protocol for low-throughput sample preparation, with a few exceptions. The protocol was followed beginning with Purify and Fragment mRNA through Normalize and Pool libraries. Libraries were indexed separately for multiplexing. Differences in the protocol and our preparation procedure were: using PCR strip tubes instead of PCR plates. The addition of step 26 in ligate adapters, transfer 20 µl of supernatant from each well to a new 0.3 ml PCR plate labeled with PCR barcode. Elute, Prime, Fragment mix was thawed and mixed into each well of the RBP plate on ice. Ligation mix was thawed on ice and mixed into each well of the ALP plate on ice.

The resulting libraries were quantified using a Thermo Scientific Nanodrop 1000 and Agilent Bioanalyzer 2100 and sent for sequencing to the National Center for Genome Resources (Santa Fe, NM). The sequencing lab further analyzed the libraries and pooled them for sequencing on a HiSeq2000, 1×100 bp reads. Reads were deposited in the NCBI sequence read archive under accession number SRP018037.

### Contig assembly and analysis

Reads were assembled in two ways; the first via assembly with Velvet with a kmer setting of 31 followed by minia, and the second by Trinity alone [41]–[43]. Contigs from the velvet-minia assembly were annotated with Blast2GO. Contigs from the Trinity assembly were used to blast protein sequences of *Rhodnius* odorant receptors and odorant binding proteins against using tblastn.

### Assembly of Orco cDNA

Reads obtained from sequencing were used to construct a blast database. The Orco protein sequence from *Rhodnius prolixus* (RPRC000476-PA) was retrieved from Vectorbase and used to search the read database. Reads aligning to the *Rhodnius* Orco sequence were then assembled with CAP3 [44].

### Phylogenetic Tree and Alignment

**Figure 1:**
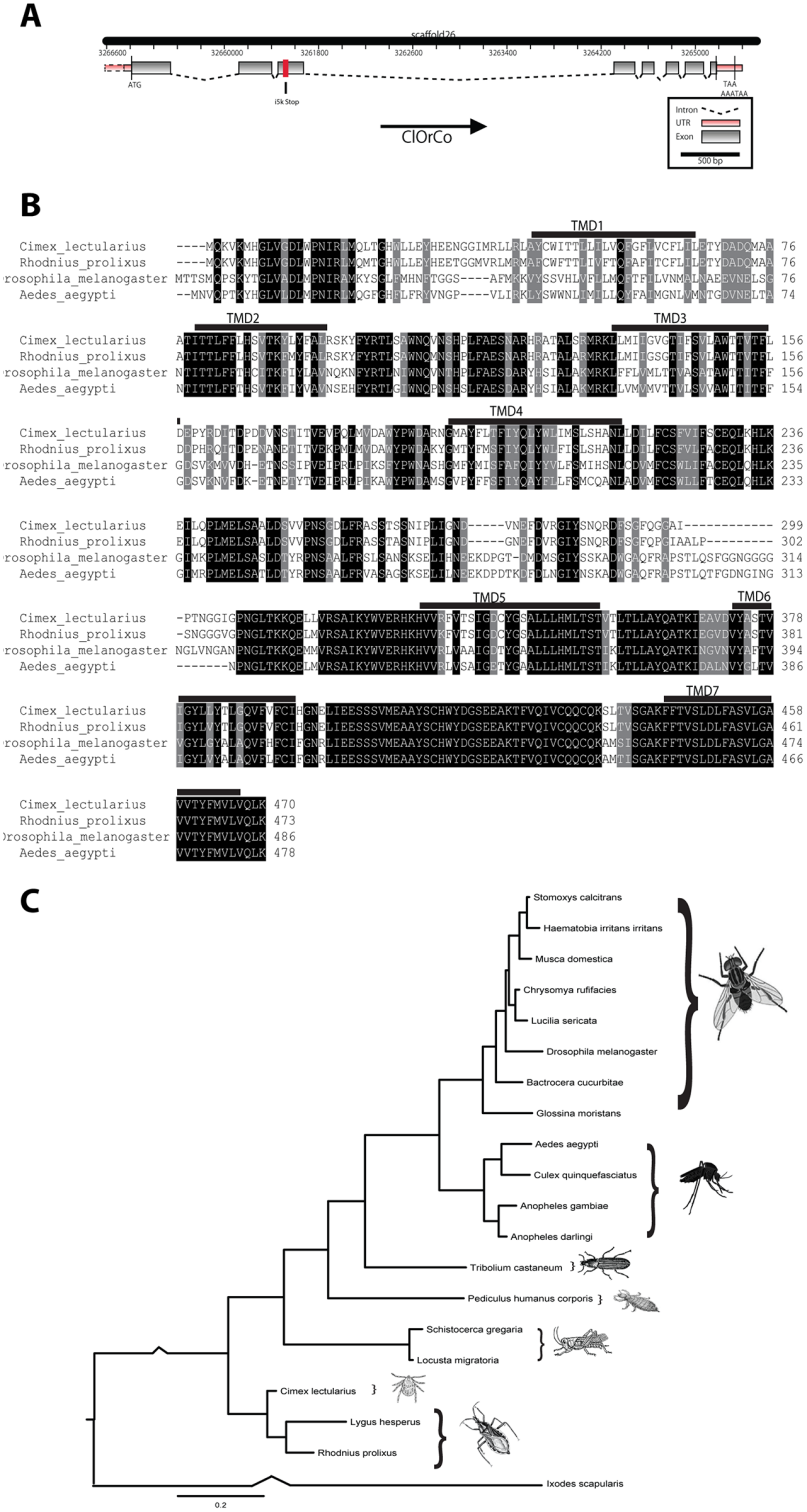
*C. lectularius* Orco gene and phylogeny. (A) Orco gene structure. Exons are boxed, introns are shown as lines. UTR – untranslated region. (B) Alignment of insect Orco proteins with transmembrane domains (TMD) indicated. (C) Phylogenetic tree.

To create figure 1B the bed bug Orco sequence we obtained from RACE PCR was aligned with Orco sequences for *D. melanogaster*, *R. prolixus* and *A. aegypti* (retrieved from Genbank) with Clustal Omega and highlighted using the sequence manipulation suite. Transmembrane domain regions were manually annotated using the regions described for *Drosophila* [45].

To create Figure 1C, the bed bug Orco sequence we obtained from RACE PCR was aligned using MUSCLE with Orco from other insect species and gustatory receptor 9 from *Ixodes scapularis*, retrieved from Genbank [46]. The aligned sequences were used to construct a neighbor-joining tree using the default options for MEGA 5.2 [47]. Accession numbers: *Schistocerca gregaria* gi|371444780, *Locusta migratoria* gi|371444778, *Lygus hesperus* gi|421991706, *Rhodnius prolixus* RPRC000476-PA (vectorbase), *Drosophila melanogaster* gi|14285640, *Aedes aegypti* gi|157111190, *Anopheles darlingi* ADAR011157 (vectorbase), *Culex quinquefasciatus* gi|167869857, *Glossina moristans* GMOY005610 (vectorbase), *Pediculus humanus corporis* gi|212509555, *Stomoxys calcitrans* gi|193795139, *Chrysomya rufifacies* gi|384503150, *Musca domestica* gi|384503152, *Lucilia sericata* gi|440631705, *Haematobia irritans irritans* gi|193795141, *Bactrocera cucurbitae* gi|302171921, *Tribolium castaneum* gi|226334904, *Ixodes scapularis* gi|215504237.

### Orco gene identification

A blast database was created using scaffolds from the i5k sequencing project for *C. lectularius*, downloaded 3/19/2013 [48]. The full length coding sequence we previously obtained was blasted against this database to identify the location of Orco and positions of exons and introns. The sequence alignment was then manually revised to account for splice site boundaries. FancyGene, the web-based software [49] was used to present gene structures on the genomic scaffold.

### Quantitative PCR

Total RNA was obtained from adult male and female bed bugs using TRIzol reagent (Ambion, Carlsbad, CA). RNA from different tissues was isolated from 30 unfed males and 30 unfed females. Relative transcript levels were quantified on an Eppendorf Mastercycler ep realplex (Eppendorf, Hamburg, Germany) using PerfeCTa SYBR Green FastMix (Quanta BioSciences, Inc., Gaithersburg, MD). Primers were as follows: qCLRPL18F: TCA TGT CCC TGA GCC ATG CAA ACT; qCLRPL18R: GCT TCG TGT GCG AGC GAG GGG; qCLOrcoF: TCA TGT CCC TGA GCC ATG CAA ACT; qCLOrcoR: TGG CAC TAC AGA ATC CAA AGC TGC A.

### Agonist and antagonist synthesis

The Orco agonist VUAA1 [50] used for these studies was synthesized following a three-step linear protocol. Commercially available methyl nicotinate was first condensed with hydrazine to generate a hydrazide intermediate that was allowed to react with ethyl isothiocyanate. The triazole thiol that formed was immediately treated with 2-chloro-N-(4-ethylphenyl)-acetamide to generate the desired agonist. Orco antagonist VU0183254 [51] was prepared in a similar manner to VUAA1 using methy-2-furoate and a 2-chloroacetamide derived from phenothiazine. Full experimental details as well as compound characterization can be found in File S1.

### Olfaction Choice Tests

**Figure 2:**
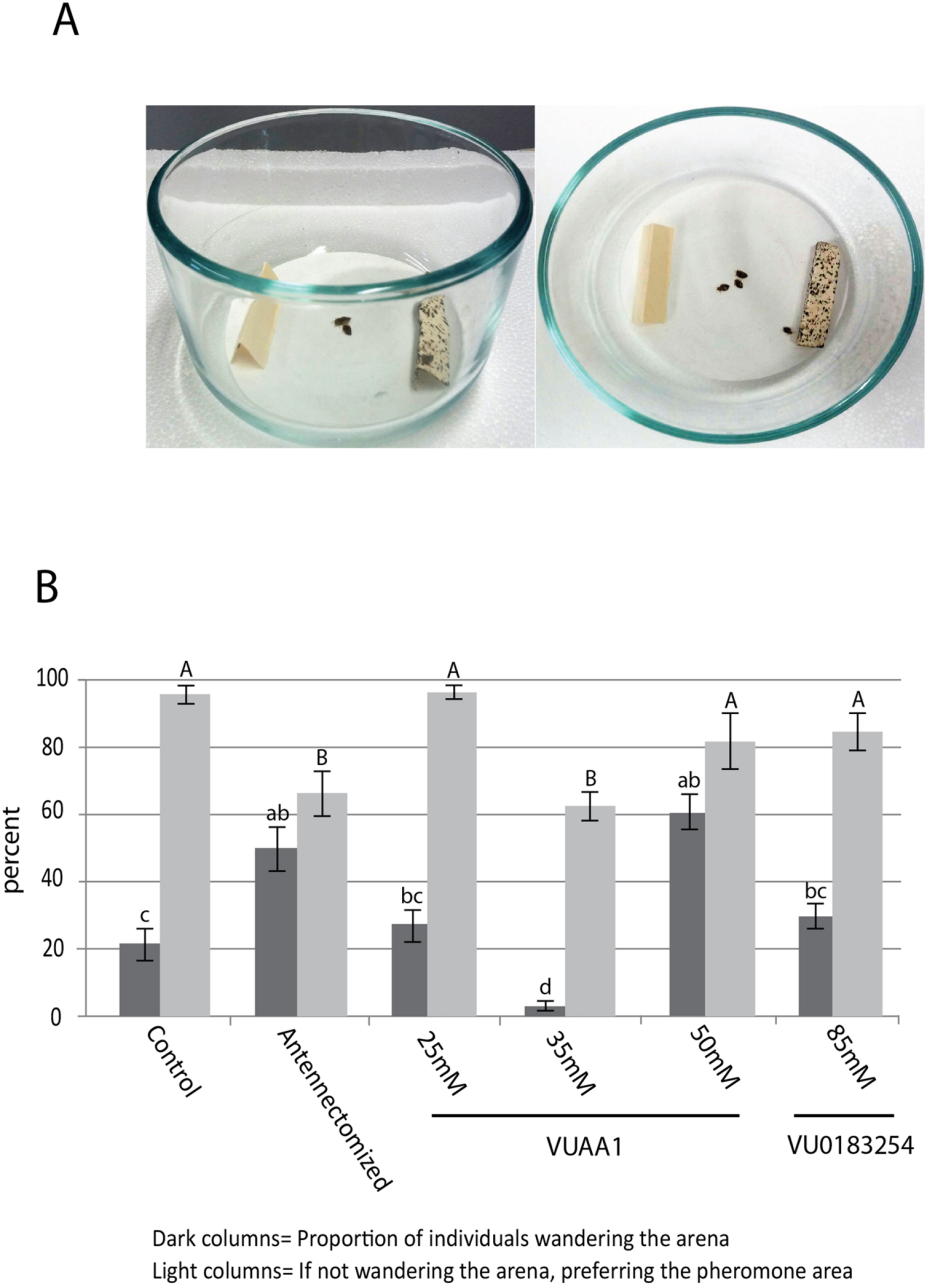
Olfaction choice assay. (A) The bed bug arenas are shown in the figure with clean paper on the left and fecal-stained pheromone paper on the right. White filter paper was taped to the bottom of the arenas. (B) Behavioral responses of bed bugs to a pheromone (fecal-stained paper) after the application of an ORCO agonist (VUAA1) and antagonist (VU0183254). The data were reported as the mean ratio (±SEM) of bed bugs attracted to the pheromone paper compared to bed bugs attracted to the pheromone paper in addition to bed bugs that remained wandering in the arena (N = 100). Lower case letters are for means comparisons between the dark bars while capital letters are means comparisons for the light bars.

Male bed bugs were placed in individual cells of 6–well cell culture plates (Corning Inc., Corning, NY). The bottoms of the cells were covered with tightly fitting filter papers. Insects were temporarily immobilized by placing plates on ice for ∼5 min. 100 male bed bugs were used for each experimental group. Topical applications (2 µl) of Orco agonist (VUAA1, 25 mM, 35 mM, and 50 mM) or antagonist (VU0183254, 85 mM) were made onto the dorsal surface of the abdomen. We chose delivery to the abdomen because we found that dipping bed bugs head first in acetone solution interfered with their sense of olfaction for several hours. Similar delivery methods have been successfully used to deliver other lipophilic molecules into insect hemolymph, for example juvenile hormone [52]. Control insects received 2 µl of acetone alone or were antennectomized. After treatment, insects were allowed to recover for 15 min. The test arenas (flat-bottomed Pyrex bowls, 12.4 cm diameter by 6.0 cm height; Corning, Corning, NY) were covered with a white filter paper (90 mm diameter; Whatman no. 2), fixed to the glass with double-sided tape. One fecal-stained paper tent (natural source of aggregation pheromone) and one clean paper tent were placed on opposite sides of the arena (See Figure 2A). All assays lasted 2 h (assays were conducted between 8 and 11 am) in dark conditions inside an environmental chamber at 25°C. Groups of 5 bed bugs (N = 100) were acclimated to the environment for 15 min by restricting them in a shell vial (21 mm diameter by 70 mm height) which was placed inverted in the center of the arena. Bed bugs were released by lifting up the shell vial. At the end of the test, the location of bed bugs resting on a tent or wandering in the arena was recorded. The antennectomized bed bugs were anesthetized with CO_2_ and all antennal segments (scape, pedicel, and both flagella segments) were removed with fine-tip forceps 24 hours before the bioassay. For the analysis, we focused our attention on two potential behavioral responses; the proportion of individuals wandering the arena and the proportion of individuals that preferred the pheromone tented area if they chose a tented area. These two responses act at different scales for the bed bugs. The first shows an ability to find the areas of interest, while the second investigates how well the pheromone signal is perceived by the treated individuals. The data were non-normal and were rank transformed and assayed with a MANOVA followed by separated ANOVAs for interpretation. A Tukey's Studentized Range (HSD) Test was conducted on the separated ANOVAs to compare between treatments [53].

### Fertility Test/Mating Bioassay

Each group of 25 virgin male bed bugs was temporarily immobilized by placing plates on ice for ∼5 min and topical applications (2 µl) of 35 mM VUAA1 or acetone were made onto the dorsal surface of the abdomen. Each individual male was placed with a recently fed female for mating. After mating was observed (at least one mating occurred within one hour) the female was transferred to individual cells of 24-well cell culture plates whose bottom was covered with tightly fitting black filter paper to facilitate egg count. After six days, number of eggs was recorded and the females were given another blood meal. Females remained at 25°C, 65±5% RH for two weeks and egg number, hatch rate and nymph viability was recorded at the end of this period. For the analysis, we removed replicates where individuals either did not blood feed at a time point or never produced eggs. This resulted in 18 replicates for the 35 mM VUAA1 treatment and 22 replicates for the acetone treatment. The data for eggs produced at day 6 and day 14 as well as the number of hatchlings at day 14 was normal and assayed with a MANOVA followed by separate ANOVAs for interpretation [53].

### Sperm motility assay

Since most bed bug sperm can be found in the seminal vesicles, we used sperm in seminal vesicles for our assay. Seminal vesicles were dissected and kept in 1× PBS on ice on the day of the experiment. Since a seminal vesicle is sufficiently large, one seminal vesicle was used for multiple assays which gave us an advantage that sperm from the same seminal vesicle could be used for different treatments (typically a half was used for control and the other half for treatment). A part of seminal vesicle was transferred to a microscope slide and 20 µL of assay buffer (125 mM NaCl, 4 mM KCl, 1 mM MgCl_2_, 1.3 mM CaCl_2_, 5 mM D-glucose, 10 mM HEPES pH 7.4 containing 10% [v/v] DMSO) with or without indicated compound was immediately put on the specimen. A coverslip was placed on the specimen and gently pressed to expose the sperm to the assay buffer. Sperm movement was observed under phase-contrast compound microscope (CX41, Olympus, Center Valley, PA) equipped with digital camera (Infinity1, Lumenera Corp., Ottawa, ON, Canada), image was video-fed by INFINITY ANALYZE software (Lunenera Corp.), and screen video was recorded by QuickTime Player (ver. 10.2, Apple Inc., Cupertino, CA). To assess drug (VUAA1) delivery to the seminal vesicles via topical application, male bed bugs were applied on abdominal tergites with 2 µL of 35 mM VUAA1 in acetone or acetone alone as control. The bugs recovered at room temperature for 5–10 min, and seminal vesicles were dissected for the assay. Experiments were repeated six to ten times. Sperm activity was assessed and scored a qualitative “motility index” (MI) similar to “activation index” described in Pitts et al. [39], where 0, no flagellum beating to 3, almost all flagella beating. Data was statistically analyzed by Mann-Whitney non-parametric U test [53].

### Histology and immunofluorescence detection

Male head and reproductive system were dissected and fixed with 4% paraformaldehyde (PFA) in PBT (0.1% Triton X-100 in PBS) overnight at 4°C. The fixed samples were washed in PBS three times for 5 min at room temperature and cryoprotected with 30% sucrose in PBT overnight at 4°C. Heads and reproductive organs were frozen in Tissue-Tek OCT compound (Sakura Fintek USA, Torrance, CA), and 10 µm thickness sections were made with Leica CM1850 cryostat microtome (Leica, Buffalo Grove, IL) and mounted on Superfrost Plus microscope slides (Fisher Scientific, Pittsburgh, PA). The slides were kept at –80°C until used. Sections were dried at room temperature for 30 min and fixed in 4% PFA in PBT for 30 min. The slides were washed in PBT three times for 5 min. After blocking in 5% goat serum in PBT (PBTG) for 1 h at room temperature, the sections were incubated in anti-*Drosophila melanogaster* Orco IC3 antibody at 1∶50 dilution in PBTG overnight at 4°C. Control slides were incubated with PBTG without antibody. Following washing in PBT three times for 5 min at room temperature, the sections were incubated with Alexa Fluor 488 goat anti-rabbit IgG (Life Technologies, Carlsbad, CA, catalog number: A-11034) at 1∶500 dilution in PBTG at room temperature for 3 h. After washing the sections in PBT three times at room temperature for 5 min, sections were mounted with ProLong Diamond Antifade Mountant with DAPI (Life Technologies), coverslips were put on the slides, sealed with nail polish and cured at room temperature in dark condition overnight. Sections were imaged by Leica TCS SP5 II confocal microscope (Leica). Control and treatment images were acquired with the same settings.

## Results and Discussion

### Sequencing

Our six libraries produced a total of 190.6 million, 100 cycle reads that passed quality control. Our assemblies generated 10,309 contigs with an N50 of 1,133 nucleotides using the velvet-minia assembly program and 219,383 contigs with an N50 of 817 using Trinity.

### Identification of putative odorant receptor/odorant-binding proteins

Nine contigs were annotated as odorant receptors and one as an odorant binding protein using blast2GO. These contigs primarily matched against *Tribolium* sequences. Analysis of our Trinity contigs using blast yielded 12 contigs which matched against 6 of 13 *R. prolixus* OBPs that are annotated in Vectorbase (Table S1). Fifty contigs matched against 16 *R. prolixus* ORs of the 111 ORs annotated in Vectorbase (Table S2).

Based on these results we hypothesize that we have identified partial cDNAs of at least six *C. lectularius* OBPs and 16 ORs. However, since some of the contigs do not overlap they could represent twelve OBPs and 50 ORs, respectively. Bed bugs seem to have less OBPs and ORs compared to *R. prolixus* (111) annotated in Vectorbase, somewhat fewer than the number of ORs in *Drosophila* (62) and *An. gambiae* (79), while significantly less than *A. aegypti* (131) [54]. While this is not a comprehensive study of olfaction-related genes in bed bugs, these results indicate that bed bugs may have a simpler system of olfactory detection than these other species.

### Orco cDNA assembly

Using BLAST, we mapped 22 reads against the *Rhodnius* Orco protein sequence. Using this in silico sequence, we assembled a putative cDNA. Primer covering the full length open reading frame were developed and used to amplify full-length Orco. The PCR product was cloned and sequenced and we published the nucleotide sequence in Genbank (GenBank accession number KM275232). *Cimex lectularius* Orco is a 451 amino acid protein, the TMHMM software [55] predicts seven transmembrane domains as has been described for the *An. gambiae* Orco, AgOR7 [56]. Mapping this cDNA against a genomic scaffold we identified from the published genome sequence shows a gene with eight exons and seven introns. A study by Pitts and coworkers shows the gene organization of *An. gambiae* and *D. melanogaster* Orco which have eight and six exons, respectively, and the three C-terminal exons with identical size [56]. Bed bug Orco has eight exons (Fig. 1A) and we did not see any similarities in exons or intron size compared to *An. gambiae* or *D. melanogaster* Orco.

After mapping our sequenced full length Orco cDNA to the i5k genome sequence, we identified a premature stop codon located in exon three of the i5k Orco sequence. This result in a predicted protein with only 209 amino acids in the i5k genome data compared to a 451aa sequence from our cloning. We hypothesize that this might be due to a sequencing or assembly mistake.

### Phylogenetic tree

The amino acid sequence of Orco is highly conserved in insects with the highest degree of identity in the C-terminus of the protein (Fig. 1B). Of the sequences we aligned with, *Cimex* Orco is most closely related to that of *Rhodnius* (89.8% identity) and other members of Hemiptera (true bugs), vs. other orders of insects. Amino acid sequence identity with *D. melanogaster* Orco was 59.1%. The phylogenetic tree shown in figure 1C is in concurrence with the results of current investigations into insect phylogenetic relationships [57].

### Orco expression in antennae and sperm

**Figure 3:**
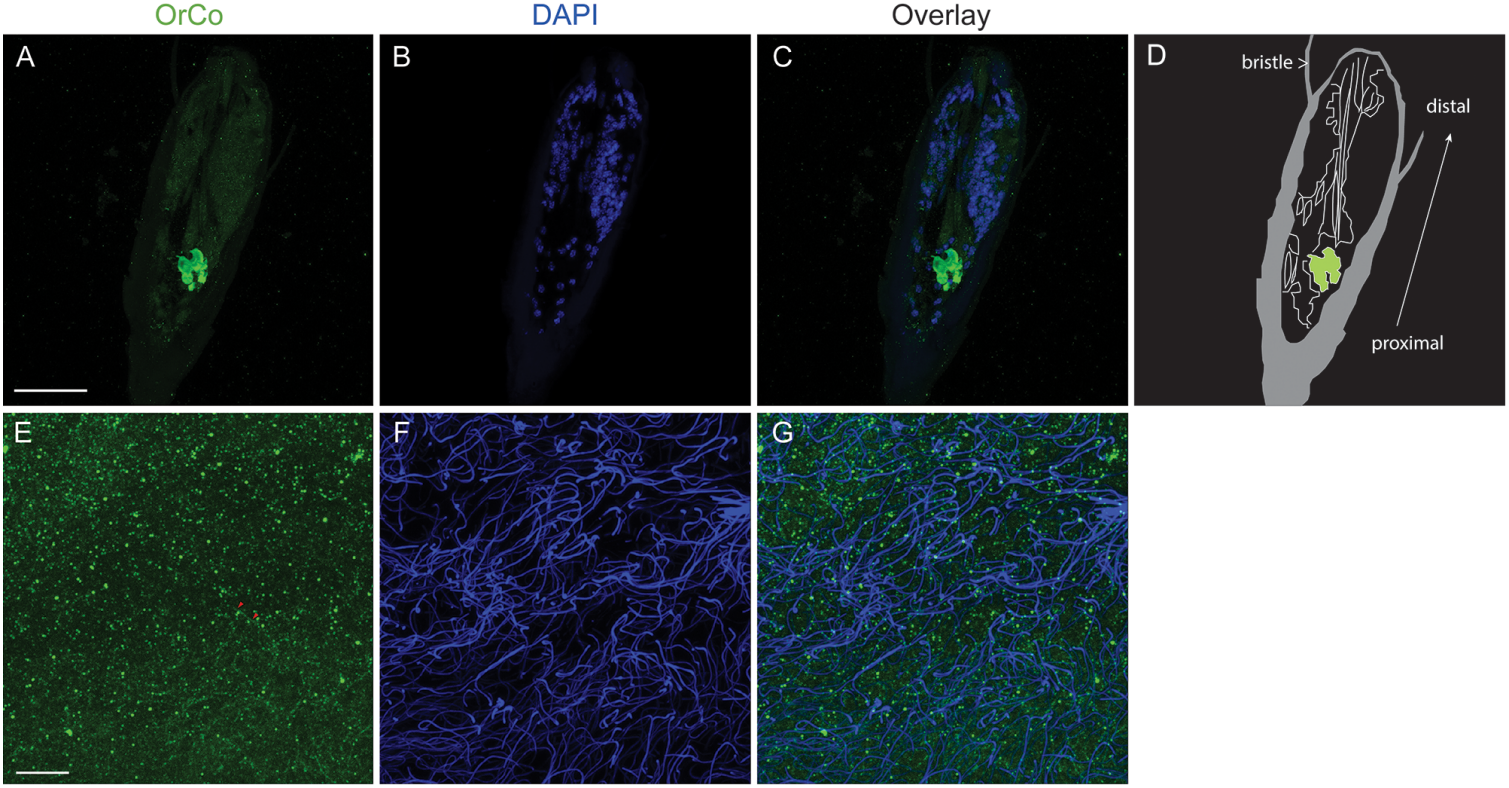
Orco protein expression in antennae and sperm - Immunofluorescence labeling with anti-*Drosophila* Orco antibody on sectioned specimens. Left panels (A and E), Orco labeling (green); center (B and F), nuclear staining with DAPI (blue); right (C and G), overlay. A–C, sections of male antennae; E–G, sections of seminal vesicles. D, Illustration of antennal section made from contrast-enhanced panel C image: grey part, cuticular structure with two serrated hairs; green, anti-Orco immune reactive cells; white lines, cellular components in antenna. White arrow indicates antennal orientation to the tip. Anterior-posterior orientation is also indicated in the figure. Scale bars: A–C, 50 µm, E–G, 10 µm. Small red arrowheads in panel E indicate a portion of flagellum.

We used an anti-*Drosophila* Orco antibody to detect Orco protein expression in antennae and sperm by immunofluorescence. Orco is expressed in the antennae, and localized in a cluster of cells that most probably represent olfactory receptor neurons (ORN) (Fig. 3A–D). Orco expression in a cluster of cells in antennae suggests that these cells are associated with specialized sensilla for olfaction such as sensilla in sensory patch that are located on the posterior part of the second antennal flagellar segment described in Olson et al. [58] which is different from the scattered Orco expression in mosquito antennae [59]. Immunofluorescence was detected from sperm in the seminal vesicles as well (Fig. 3E–G). Hemipteran sperm cells are known to have elongated nuclei (25–50 µm), which is also the case for *C. lectularius* sperm cells [60], [61]. Orco staining did not co-localize with nucleus and is present in punctate distribution along the tail as has been shown in *An. gambiae* (Fig. 1 in [62]). For more details, larger figures (Fig. S1) and 3D movie (Video S1) are provided as supporting information.

### Orco expression in different tissues

**Figure 4:**
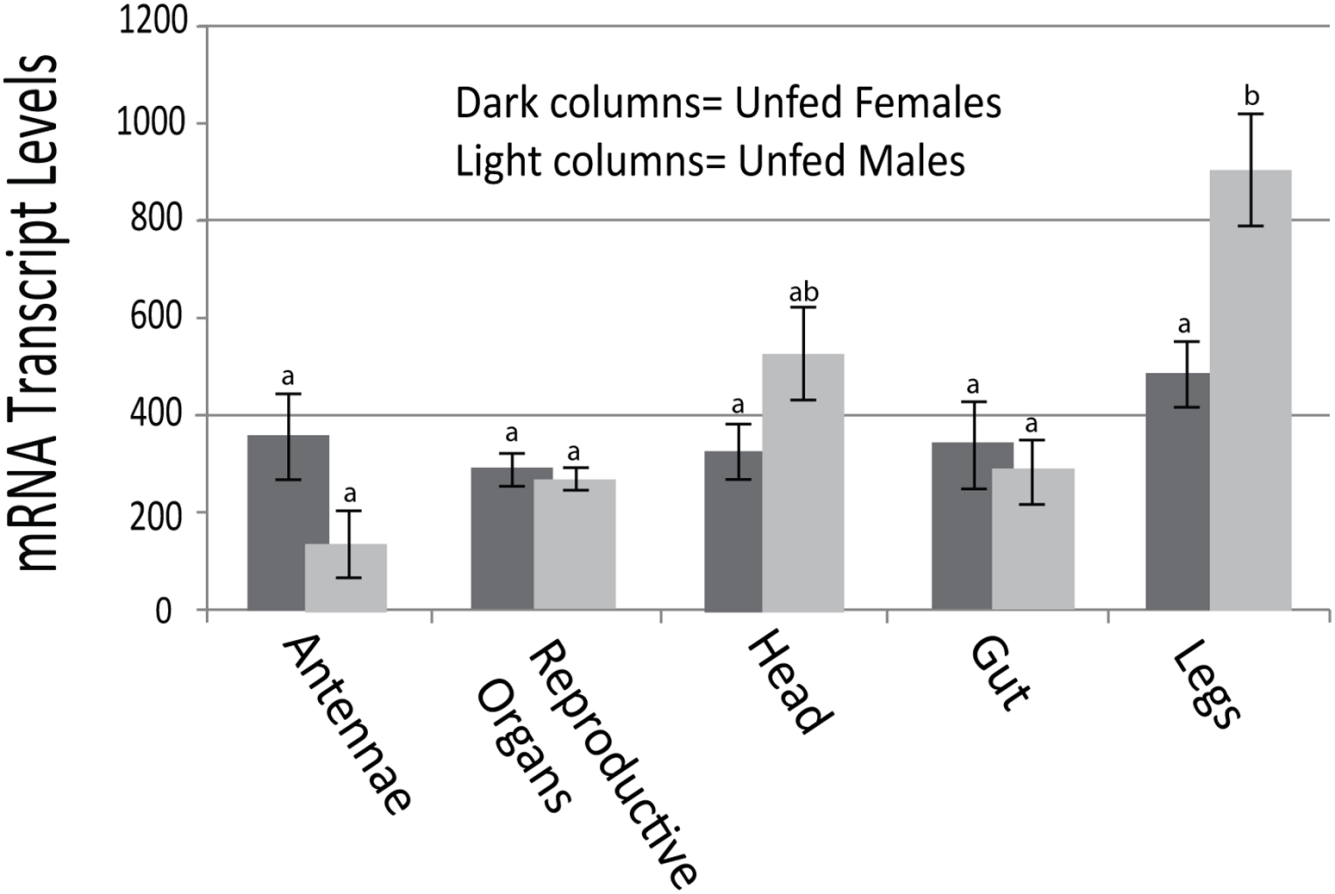
Orco tissue expression. Expression was assayed using q-RT-PCR. Expression values were normalized with the control expression data from the same samples. The results shown are representative of three separate repeats with similar results. RNA was isolated from organs/body parts of unfed adult female bed bugs (dark-shaded columns) and unfed adult male bed bugs (light-shaded columns). The means were separated by the Tukey-Kramer HSD (p<0.05). Means which share the same letter are not significantly different.

Quantitative-RT-PCR was used to determine Orco expression in unfed adult male and female bed bugs. Orco is highly expressed in the antennae and legs of the female bed bugs with slightly reduced expression in reproductive organs, head, and the gut (Fig. 4). There was higher Orco expression in male legs and head than the female bed bugs, but lower expression of Orco in the male antennae than the female. Expression in the male reproductive organs and gut closely resembles female expression patterns. Orco expression in male legs is consistent with other studies which found expression of odorant and gustatory receptors in male *Drosophila* legs [60], [63]. In *An. gambiae*, another holometabolous insect, G-Protein Receptor (GPR) odorant receptors were found to be expressed in olfactory tissues and legs [61], [64]. Orco transcripts have been shown to be expressed in male and female antennae of the hemimetabolous insect, *Locusta migratoria*. Mouthparts, tarsi and wings also show expression of Orco, demonstrating a ubiquitous expression in varying tissues throughout *L. migratoria* [45]. Similar expression was shown in figure 4, as overall expression in different body parts of the bed bugs had no significant difference from one another except for male legs.

### Effect of Orco agonist and antagonist on bed bug pheromone-induced aggregation behavior

The chemicals VUAA1 and VU0183524 have been demonstrated to be agonist and antagonist, respectively, for Orco in *Drosophila melanogaster* and mosquito species (*An. gambiae* and *Ae. aegypti*) and interact with these insect odorant receptors [37], [38], [65]. VUAA1 was effective in a mobility bioassay in mosquito larvae and the activation effect was dependent on Orco expression [50]. To our knowledge these agents have not been tested in hemipterans.

Bed bug arena bioassays were conducted to determine the bed bug's ability to locate a pheromone after the application of an Orco agonist and antagonist to the abdomen. The treatments resulted in a significant effect (F_10,212_ = 16, P<0.001) with shifts in both the proportion of individuals making a choice (F_5,107_ = 19, P<0.001) and the proportion of individuals preferring the pheromone area having made a choice (F_5,107_ = 14, P<0.001). At the lowest doses of VUAA1 (25 mM), there was not a significant difference present as compared to the control in either of the two responses (Fig. 2B). Surprisingly, at 35 mM of VUAA1, more bed bugs made a choice and that choice resulted in fewer individuals preferring the pheromone area as compared to the control and the 25 mM of VUAA1 treatment. The 50 mM VUAA1 treated bed bugs had more individuals not making a choice as compared to the control and 25 mM of VUAA, but those individuals that did choose showed similar preference for the pheromone area (Fig. 2B). The 85 mM VU0183254 antagonist treated bed bugs showed no significant difference in choice or preference for the pheromone area as compared to the control and 25 mM of VUAA1 treatment. The antennectomized bed bugs showed an increase in the proportion of individuals failing to choose an area as well as a lower proportion of individuals preferring the pheromone area as compared to the control and 25 mM VUAA1 treatment. Interestingly their responses match some of the effects of the Orco agonist and antagonist treatments (Fig. 2B). Results from our antennal manipulation experiments confirm that sensory organs and their associated odorant binding proteins and odorant receptors located on the antennae are essential for detection of pheromone-stained papers. Our results also show that the Orco agonist VUAA1 changed bed bug pheromone-induced aggregation behavior, but only at specific concentrations. Those changes can affect both the proportion of individuals choosing an area as well as how attracted those individuals are to a pheromone cue. It is unclear if the effects we found are due to over activation of Orco or if another mechanism is involved. The absence of effect at the lowest concentrations of the agonist and for the antagonist might be due to our very inefficient acetone-based delivery system. Both chemicals show little solubility in water so changes in structure or an optimized delivery system might be necessary to develop these compounds into effective repellents.

### Effect of Orco agonist on bed bug mating efficiency

**Figure 5:**
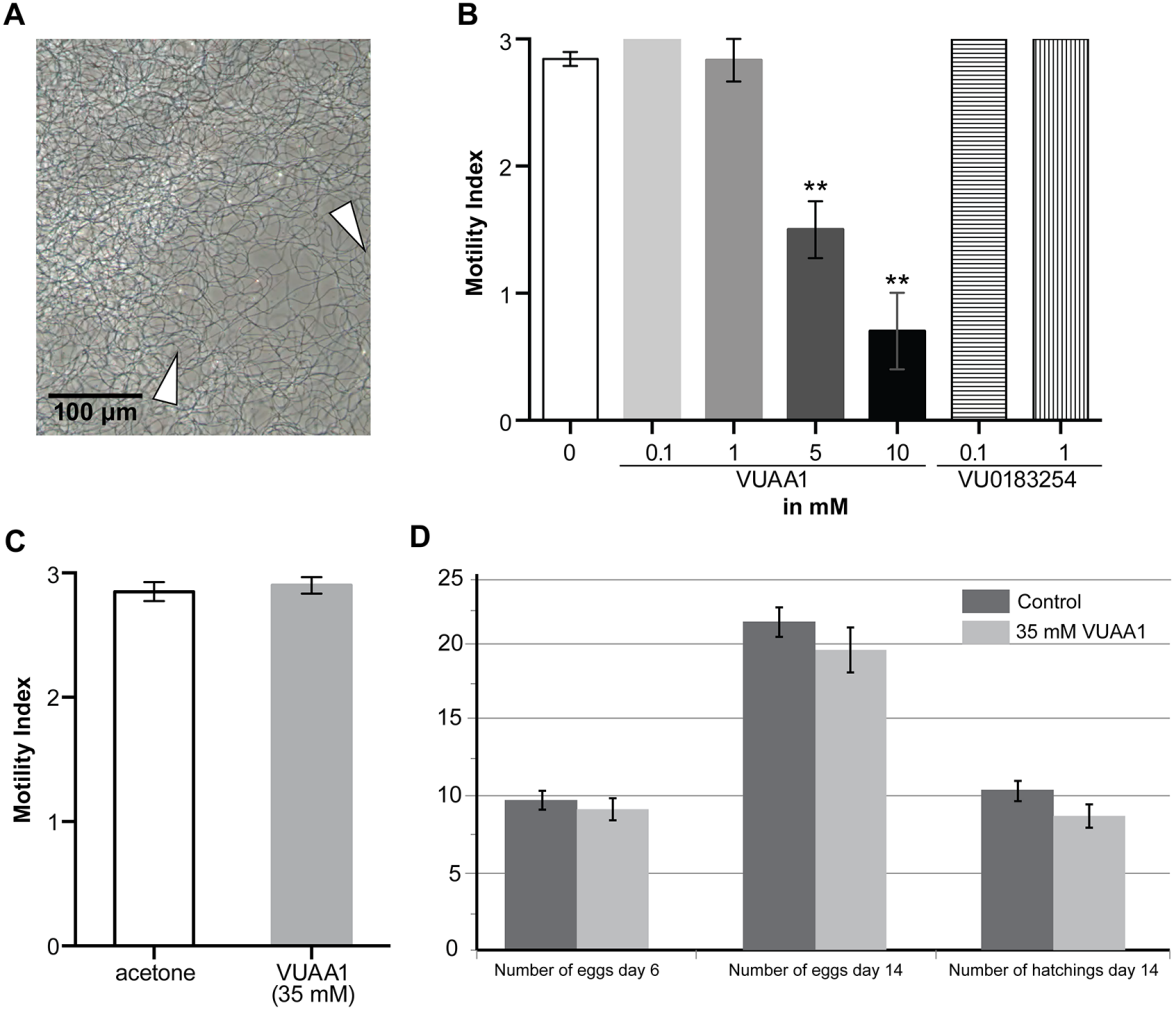
Sperm motility modulation and effects on egg production and hatching by Orco agonist and antagonist. (A) Phase-contrast micrograph of the bed bug sperm. Black hair-like filamentous are sperm [a linear segment is indicated by a pair of white arrowheads, see also Video S2 (control) and Video S3 (sperm treated with VUAA1). (B) Motility indices (vertical axis) of the bed bug sperm for indicated concentrations of compounds tested (horizontal axis). (C) Sperm motility from the bugs topically applied with VUAA1 (2 µL of 35 mM in acetone) or acetone alone as control. (D) Egg production and hatching success for the control and Orco agonist (35 mM VUAA1). Values are in mean ± SEM. **indicates statistical significance at *p*<0.01 in comparison to control by non-parametric Mann-Whitney U test for B and C. No significant difference was detected by MANOVA for D.

The application of (2 µl) of 35 mM VUAA1 or acetone did not collectively alter mating efficiency (F_3,36_ = 1.6, P = 0.21). The number of eggs produced (F_1,38_ = 0.3, P = 0.61; F_1,38_ = 1.6, P = 0.21 respectively) and the number of hatched nymphs (F_1,38_ = 2.7, P = 0.11) was always similar between the two treatments (Fig. 5D).

### Effect of Orco agonist and antagonist on bed bug sperm

Pitts and colleagues showed that VUAA1 and VU0183524 modulated activity of mosquito sperm cells that express Orco protein [39]. We tested both compounds to assess their modulating effects on motility of the bed bug sperm. Unlike mosquito sperm cells, bed bug spermatozoa are active once they are dissected out of seminal vesicles into the assay buffer as long as they remain in aggregates (Video S2). At 5 mM of VUAA1, sperm activity was reduced significantly, and at 10 mM sperm were almost completely halted (Fig. 5B). This phenomenon was not observed at lower dosage of VUAA1 tested. Unfortunately, VU0183524 could not be used at higher than 1 mM since its solubility in assay buffer was exceedingly lower than VUAA1 and precipitated as crystals at such concentrations. Inactivation of sperm with the Orco agonist VUAA1 (Video S3) suggests that Orco has functions in sperm activity although the mechanism seems to be different from that in mosquitoes. Topical application of VUAA1 on male abdomen did not change sperm motility in the seminal vesicles (Fig. 5C), which explains why we did not see differences in mating efficiency and female reproductive capability (Fig. 5D). The fact that neither sperm motility nor mating efficiency was affected by our treatments suggests that the drug delivery to the seminal vesicle was ineffective. Despite these negative results we suggest that with a more effective drug delivery system it might be possible to sterilize bed bug males using Orco inhibitors. Further studies are needed to elucidate the mechanisms of sperm motility control via Orco.

## Conclusions

Most blood sucking arthropods are dependent on their olfactory system to find a host and acquire and blood meal. Olfactory cues from hosts are sensed through the activation of odorant receptors localized on the antenna, the major olfactory organ in bed bugs. It is therefore anticipated that a greater understanding of the olfactory process may contribute the development of methods that interfere host finding including the use of repellents. Morphological studies of bed bug’s antenna have shown 3 different sensillum types in antenna that respond electrophysiologically to odorants previously reported to be bioactive in various haematophagous arthropods [34], [66].

In this study we present a series of experiments designed to identify odorant binding proteins, odorant receptors and odorant receptor co-receptor (Orco) from sensory organs of a medically important urban pest, *C. lectularius*. We identified a relatively low number of OBPs and ORs which correlates well with the low number of olfactory sensilla present on the antenna of bed bugs. A bed bug’s antenna has only 44 olfactory sensilla, 50-fold fewer than the blood-sucking hemipteran *Triatoma infestans* (vector of Chagas disease) [67]. The low number of these structures in bed bugs has been associated with their closer association to their hosts compared with the more “adventurous” *T. infestans* that has a broader host spectrum ranging from rattlesnakes to birds to humans [67].

We have described and characterized the first time odorant receptor co-receptor (Orco) in a hemimetabolous insect. The striking conservation of Orco observed across insect taxa is an important feature when proposing a role for these genes in olfactory signaling in insects. We also observed expression of Orco in various bed bug organs, which indicate that Orco is functionally required for a broad range of chemosensory driven behavior. We evaluated the potential role of Orco in sperm motility with an Orco agonist and an Orco antagonist. Assays with the agonist VUAA1 showed that, contrary to what occurs in mosquitoes, sperm motility of bed bug males is dramatically reduced, however no effect on fecundity was observed. The mechanism for reduction of sperm motility is unclear and further work with other agonists and antagonists is needed to fully elucidate the role of Orco in bed bug sperm function and explore new ways to interfere with bed bug reproduction. We also explored the potential of an agonist an antagonist for disrupting aggregation behavior in bed bugs. Our results showed that the Orco agonist VUAA1 changed bed bug pheromone-induced aggregation behavior. The results of this study suggest that bedbug Orco is a high-value target for the development of novel behavior modifiers for bedbug control.

## Supporting Information

Figure S1 **These panels are larger version of** **Fig. 3E–G** **. A, B, C correspond to** **Fig. 3E, F, G** **, respectively.** Red arrowheads in E indicate a portion of a flagellum. Scale bar, 10 µm.
(TIF)

Table S1 **Contigs of OBP genes potentially involved in olfaction.**
(XLSX)

Table S2 **Contigs of OR genes potentially involved in olfaction.**
(XLSX)

File S1 **Synthesis of VUAA1 and VU0183524.**
(DOCX)

Video S1 **3D projection of multiple optical sections of seminal vesicle shown in** **Fig. 3** **.** Continuous Orco labeling (green) along flagella is visible.
(MOV)

Video S2 **Control bed bug sperms in assay buffer.**
(MOV)

Video S3 **Bed bug sperms treated with 10 mM VUAA1 in assay buffer.**
(MOV)
