# Correction: The Odorant Receptor Co-Receptor from the Bed Bug, *Cimex lectularius* L

**DOI:** 10.1371/journal.pone.0119059

**Published:** 2015-03-25

**Authors:** The PLOS ONE Staff

There is an error in one of the primer sequences in the last sentence under the subheading Quantitative PCR in the Materials and Methods section. The first primer sequence should be: qCLRPL18F GGA AGA GGA ATG CTC GGG AGG CTG T. The correct sentence is: Primers were as follows: qCLRPL18F GGA AGA GGA ATG CTC GGG AGG CTG T; qCLRPL18R: GCT TCG TGT GCG AGC GAG GGG; qCLOrcoF: TCA TGT CCC TGA GCC ATG CAA ACT; qCLOrcoR: TGG CAC TAC AGA ATC CAA AGC TGC A.
