# Supplementary figures and images for: The Odorant Receptor Co-Receptor from the Bed Bug, Cimex lectularius L
Source: PLoS One. 2014 Nov 20;9(11):e113692. doi: 10.1371/journal.pone.0113692 (PMC4239089; doi:10.1371/journal.pone.0113692)

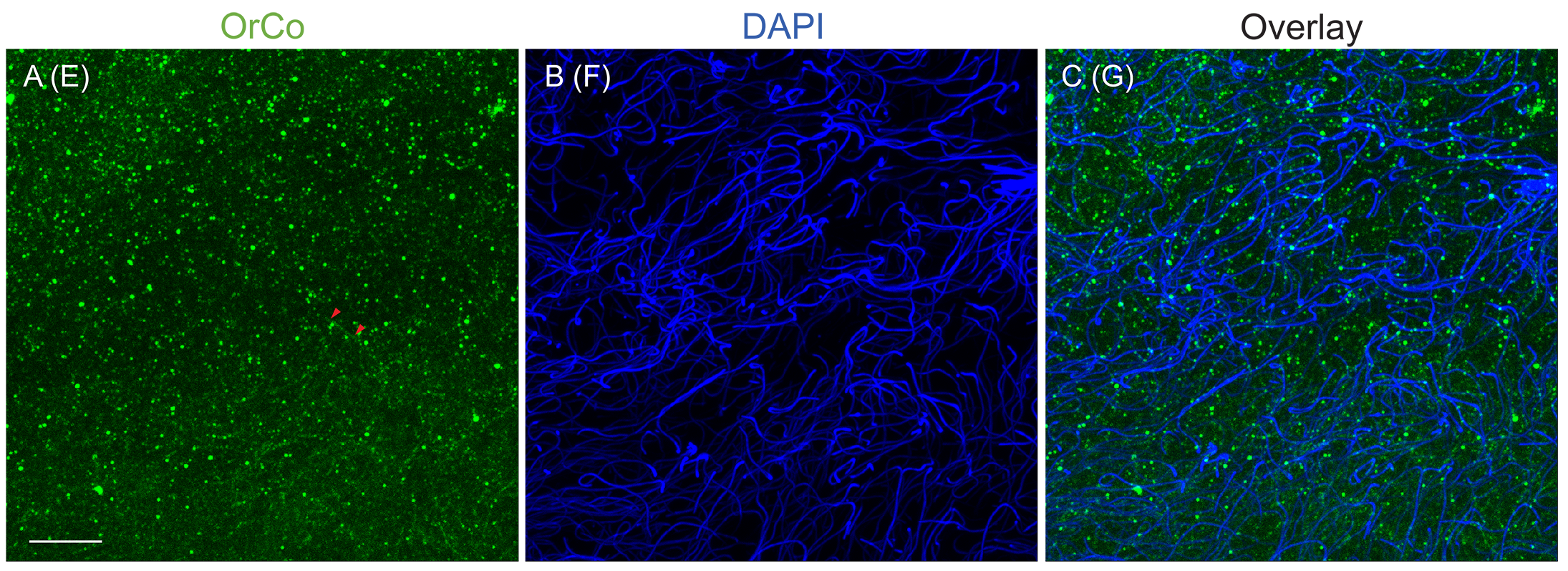

Supplement: Figure S1 — These panels are larger version of Fig. 3E–G . A, B, C correspond to Fig. 3E, F, G , respectively. Red arrowheads in E indicate a portion of a flagellum. Scale bar, 10 µm. (TIF) [file pone.0113692.s001.tif]
