# Supplementary material for: The Odorant Receptor Co-Receptor from the Bed Bug, Cimex lectularius L
Source: PLoS One. 2014 Nov 20;9(11):e113692. doi: 10.1371/journal.pone.0113692 (PMC4239089; doi:10.1371/journal.pone.0113692)
Supplement: File S1 — Synthesis of VUAA1 and VU0183524. (DOCX) [file pone.0113692.s004.docx]

**Scheme 1. Synthesis of ORCO Agonist VUAA1 (1)**

**Scheme 2. Synthesis of ORCO Antagonist VU0183254 (2)**

**Experimental Procedure and Characterization**

Unless otherwise noted, reactions were performed in flame-dried glassware under an atmosphere of dry nitrogen. Reaction solvents (CH_2_Cl_2_, THF, and Et_2_O) were purified before use in a solvent purification system under a flow of dry nitrogen. All other solvents and reagents were purchased from commercial suppliers and used as received, unless otherwise specified. Thin-layer chromatography (TLC) was performed using plates precoated with silica gel 60 Å F- 254 (250 μm) and visualized by UV light, KMnO4, or anisaldehyde stains, followed by heating. Silica gel (particle size 40-63 μm) was used for flash chromatography. ^1^H and ^13^C NMR spectra were recorded at 300 MHz and 75 MHz or at 400 MHz and 100 MHz, respectively, and are reported relative to residual solvent peak (δ 7.26 and δ 77.0 for ^1^H and ^13^C in CDCl3. Data for ^1^H NMR spectra are reported as follows: chemical shift (δ ppm) (multiplicity, coupling constant (Hz), integration). Spectra obtained are described using the following abbreviations: s = singlet, d = doublet, t = triplet, q = quartet, m = multiplet.

**ORCO Agonist VUAA1 (1)** was prepared following a known literature procedure.^1^ To a solution of methyl nicotinate (0.20 g, 1.5 mmol) in ethanol (1.0 mL) was added a solution of hydrazine hydrate (1.0 M in water, 0.73 mL, 15.0 mmol). The reaction mixture was then allowed to warm to a gentle reflux over the course of 2 h and then cooled to rt. At this stage, the contents of the flask were diluted with methanol and concentrated *in vacuo*. The crude product was purified via flash chromatography (20% Methanol / 80% CH_2_Cl_2_) to afford 0.081 g (40%) of isonicotinohydrazide as a yellow oil. This compound was then dissolved in 1.0 mL of ethanol and ethyl isothiocyanate (0.06 mL, 0.70 mmol) was added in a single portion. The contents of the flask were then brought to a gentle reflux and allowed to stir at 80 °C overnight before being concentrated *in vacuo*. The crude white solid that resulted was dissolved in 10 mL of water, treated with K_2_CO_3_ (0.09 g, 0.70 mmol), and vigorously stirred at 100 °C overnight. After cooling to rt, the contents of the flask were diluted with methanol and concentrated *in vacuo*. The crude product was purified via flash chromatography (15% Methanol / 85% CH_2_Cl_2_) to afford 0.081g (66%) of triazole thiol as a white solid.

In a separate flask, 4-ethylaniline (0.07 mL, 0.56 mmol) and triethylamine (0.08, 0.56 mmol) were dissolved in dichloromethane (5.6 mL). This solution was then treated with chloroacetyl chloride (0.05 mL, 0.56 mmol) and was allowed to stir at room temperature for 2 h before being concentrated, redissolved in dry aceteonitrile (5.6 mL), and treated sequentially with triazole thiol (0.077 g, 0.38 mmol) and Cs_2_CO_3_ (0.245 g, 0.75 mmol). The reaction mixture was allowed to stir at room temperature overnight before being concentrated and purified via flash chromatography (15% Methanol / 85% CH_2_Cl_2_) to afford 0.108 g (78%) of ORCO agonist VUAA1 (**1**) as a white solid. ^1^H and ^13^C data were in agreement with literature values.^1^

**Triazole thiol 5** was prepared following a known literature procedure.^1^ To a solution of methy 2-furoate (0.2 mL, 1.87 mmol) in ethanol (1.0 mL) was added a solution of hydrazine hydrate (1.0 M in water, 0.94 mL, 18.7 mmol). The reaction mixture was then allowed to warm to a gentle reflux over the course of 2 h and then cooled to rt. At this stage, the contents of the flask were diluted with methanol and concentrated *in vacuo*. The crude product was purified via flash chromatography (10% Methanol / 90% CH_2_Cl_2_) to afford 0.215 g (91%) of furan-2-carbohydrazide as a yellow oil. This compound was then dissolved in 2.0 mL of ethanol and ethyl isothiocyanate (0.18 mL, 2.05 mmol) was added in a single portion. The contents of the flask were then brought to a gentle reflux and allowed to stir at 80 °C overnight before being concentrated *in vacuo*. The crude white solid that resulted was dissolved in 10 mL of water, treated with K_2_CO_3_ (0.283 g, 2.05 mmol), and vigorously stirred at 100 °C overnight. After cooling to rt, the contents of the flask were diluted with methanol and concentrated *in vacuo*. The crude product was purified via flash chromatography (2% Methanol / 98% CH_2_Cl_2_) to afford 0.327 g (98%) of triazole thiol **5** as a white solid. ^1^H NMR (δ, ppm, CD_3_OD, 400 MHz) 7.80 (dd, *J* = 0.88, 1.91 Hz, 1H), 7.09 (dd, *J* = 0.73, 3.52 Hz, 1H), 6.68 (dd, *J* = 1.91, 3.52 Hz, 1H), 4.35 (q, *J* = 7.19, 14.38 Hz, 2H), 1.34 (t, *J* = 7.04, 3H).

**ORCO antagonist VU0183254 (2)**.^2^ Phenothiazine (2.0 g, 10.0 mmol) and chloroacetyl chloride (0.96 mL, 12.04 mmol) were dissolved in dry toluene (20 mL) and warmed at a gentle reflux for 2 h before being concentrated and redissolved in dry aceteonitrile (19.0 mL) and treated sequentially with triazole thiol **5** (0.3731 g, 1.91 mmol) and Cs_2_CO_3_ (1.25 g, 3.82 mmol). The reaction mixture was allowed to stir at room temperature overnight before being concentrated and purified via flash chromatography (5% Methanol / 95% CH_2_Cl_2_) to afford 0.608 g (73%) of ORCO antagonist VU0183254 (**2**) as a white solid. ^1^H NMR (δ, ppm, DMSO, 400 MHz) 7.04 (dd, *J* = 0.73, 1.91, 1H), 6.82 (d, *J* = 7.48 Hz, 2H), 6.70 (dd, *J* = 1.47, 7.63 Hz, 2H), 6.54 (td, *J* = 1.47, 7.48, 9.10 Hz, 2H), 6.45 (td, *J* = 1.47, 7.63, 8.95 Hz, 2H), 6.16 (dd, *J* = 0.73, 3.52 Hz, 1H), 5.82 (dd, *J* = 1.76, 3.52 Hz, 1H), 3.51 (bs, 2H), 3.22 (q, *J* = 7.19, 14.38 Hz, 2H), 0.33 (t, *J* = 7.19, 3H) ; ^13^C NMR (δ, ppm, CDCl_3_, 400 MHz) 166.5, 150.3, 147.7, 144.0, 142.5, 138.1, 128.2, 127.5, 111.9, 111.8, 40.6, 38.0, 15.6.

1. Taylor, R. W.; Romaine, I. M.; Liu, C.; Murthi, P.; Jones, P. L.; Waterson, A. G.; Sulikowski, G. A.; Zwiebel. *ACS Chem. Biol*. **2012**, *7*, 1647-1652.
2. Belei, D.; Dumea, C.; Samson, A.; Farce, A.; Dubois, J.; Bîcu, E.; Ghinet, A. *Bioorganic Med. Chem. Lett*. **2012**, *22*, 4517-4522.
